# The Risk of T2DM in College Women: The Predictive Power of Financial versus Residential Status in a Cross-Sectional Pilot Study in Turkey

**DOI:** 10.3390/bs12090309

**Published:** 2022-08-26

**Authors:** Aleksandra S. Kristo, Çağla Pınarlı, Anita H. Kelleher, Stefanos L. Kucuknil, Angelos K. Sikalidis

**Affiliations:** 1 Department of Food Science and Nutrition, California Polytechnic State University, 1 Grand Ave., San Luis Obispo, CA 93407, USA; 2 Department of Nutrition and Dietetics, Istanbul Yeni Yuzyil University, Yilanli Ayazma Yolu No 26, Istanbul 34010, Turkey; 3 Balıklı Greek Hospital, Physiotherapy Clinic, Belgrad Kapi Yolu No 2, Istanbul 34020, Turkey; 4 Center for Health Research, California Polytechnic State University, 1 Grand Ave., San Luis Obispo, CA 93407, USA

**Keywords:** dietary habits, type 2 diabetes risk, income level, female university students, Turkey

## Abstract

*Aim:* This study aimed to investigate the relationships between dietary habits, income levels and type 2 diabetes mellitus (T2DM) risk in Turkish female university students who are living with their family or in the dormitory. *Materials and Methods:* This work was a cross-sectional pilot study conducted during December 2016–January 2017 in Istanbul Yeni Yuzyıl University. A survey was administered to 100 female students, 60 living with their family and 40 in dormitories. Income level was determined based on TURKSTAT 2015 percentiles. T2DM risk was determined using the Finnish Diabetes Association Type 2 Diabetes Risk Assessment Form (FINDRISC). Food frequency questionnaire and 24 h dietary recall results were analyzed by the diet analysis software Beslenme Bilgi Sistemi (BeBiS), specially developed for Turkey. *Results:* Results indicated inadequacies and imbalanced nutrition among female college students overall. Notably, there was a statistically significant higher diet quality for the students living with their families compared to those living in dormitories. Income level was consistently positively associated with better nutritional outcomes, while negatively associated with T2DM risk, but interestingly, only in the case of students living in the dormitory and not for those living with family. *Conclusions:* Our findings indicated that financial status, rather than living in the dormitory versus with family, is positively associated with increased T2DM risk as assessed via FINDRISC among Turkish female college students. This study’s results indicate a potential need for educational programs and nutritional support for students, particularly those living away from family.

## 1. Introduction

Human health and well-being are affected by a broad variety of factors such as nutrition and diet, genetic background, socio-psychological, environmental factors, and others. Nutrition is a key factor especially in terms of acting in preventing obesity as well as an entire host of non-communicable diseases (NCDs) such as cardiovascular disease (CVD), diabetes and many cancers [1]. NCDs are on the rise affecting a large segment of the global population regardless of gender, race, religion, and national origin. Interestingly, NCDs constitute the cause of death for more than half of the global deaths in the recent years. More specifically, the World Health Organization (WHO) reports that 69% of all deaths were attributed to NCDs in 2011 while this trend is projected to increase further [2,3]. As childhood obesity and increasingly earlier ages in diabetes diagnosis are seen more frequently, further concerns about public health are raised. The WHO and other stakeholders underline the need for this alarming trend to be addressed. Focus is given on behavioral factors linked to nutrition and diet, which include nutrition knowledge and the environment (family/social, socioeconomic status, accessibility of quality food, lifestyle). Furthermore, the importance of interventions at the earlier stages of life and independent living, such as the college years, is highlighted [4].

Being a fast-developing economy, Turkey, is simultaneously facing issues of nutrition deficiencies along with rising levels of chronic disease, mostly of metabolic and cardiovascular nature and diabetes, related to rapid urbanization and adoption of a Western diet and lifestyle model [4]. According to the Turkish Diabetes Foundation official announcement in 2016, approximately 15% of Turkey’s adult population has diabetes and the number of patients with diabetes is rapidly increasing. More specifically, the number of patients with diabetes has almost doubled in the last 10 years, increasing from 7.6% to 13.4%, while a significant number of people are not aware that they are diabetic [5]. This situation poses serious concerns and questions as to what points of intervention may be identified for a more effective potential reversal of this trend to be seen. Early years of independent living have been identified as such in the Turkish setting particularly [4,5].

Inadequate and imbalanced nutrition constitutes a very important multidimensional problem. According to some research, university students do not receive adequate and/or balanced diets [6,7,8,9]. Additionally, it has been reported that there is a higher likelihood for depression development in university students with high inflammatory load diets [10]. According to WHO World Mental Health Surveys there is an increased risk for mental disorders, stress, and anxiety among university students [11] which may well generate a vicious cycle with comfort food being sought thus deteriorating dietary habits and potentially undermining health. Work has revealed that chronic stress, low-grade chronic inflammation and body composition are interrelated and potentially affected negatively by imbalanced dietary intakes in university students [12]. Furthermore, the inadequate/imbalance diet problem often observed in college students may be more exacerbated in those living in dormitories due to practical and logistical challenges in addition to stress [13,14]. Other reasons contributing, not exclusively though, to nutritional problems include inadequate education and low economic status. Nutrition in youth, including early college years, is very important for health status, academic success and prevention of chronic diseases such as obesity, CVD, cancer, and diabetes in the long term.

In the work presented herein, we were interested to see whether and how the living status has an impact on the dietary habits of university students, and how these results affect the risk of T2DM. More specifically, this work aimed to investigate the interrelationship between income level, dietary habits and type 2 diabetes mellitus risk on Turkish female university students who are living with their family or in university dormitories. We hypothesized that students at lower income would exhibit higher risk for T2DM, higher BMI and lower quality dietary habits compared to their peers of higher economic status. We further hypothesized that there would be a positive association between living in the dormitory and risk of T2DM in this cohort of Turkish female students. Conducting this work in this particular population, is important since our cohort has an age that signifies formative years, whereby eating habits can have a profound effect on risk for chronic diseases in the future as the population ages.

## 2. Materials and Methods

### 2.1. General Characteristics

All participants were female adults enrolled as full-time students. The study assessed 100 female students, 60 of whom were living with their family while the remaining 40 were living in dormitories. Inclusion criteria were as follows: being female in the 19–23 years old age range (typical college student age), not being diabetic, no known allergies and medical conditions, no medication, and no regular nutrition supplements intake. Food frequency questionnaires were administered and 24 h recalls were conducted with all participants through face-to-face interviews with the same female trained registered dietitian nutritionist. The following parameters were considered: age, sex, living status, diabetes history in the family, hypertension history, physical activity level, income level, smoking status, alcohol consumption status and anthropometrics (i.e., weight, height, BMI, and waist circumference). All eligible participants provided their informed consent in writing prior to their enrollment in the study.

### 2.2. Food Frequency Questionnaire (FFQ)

A standardized Food Frequency Questionnaire (FFQ) validated for the Turkish population [15], was used to derive information on food consumption patterns of participants. This FFQ includes major food groups (milk/dairy products, meat/egg/legumes, fruits, vegetables, grains, sugar/desserts). Frequency was assessed as follows: every day, 1–2 times a week, 3–4 times in a week, 5–6 times in a week, 2 times a month, 1 time a month, and never consumed. Amount of food consumed was also assessed.

### 2.3. Twenty-Four-Hour Dietary Recall

A standard 24 h recall was applied to all participants by the same trained female registered dietitian nutritionist. Data from the 24 h dietary recall were analyzed with Beslenme Bilgi Sistemi (BeBiS) (in English: Nutritional Information System) version 8.0 (2017) developed by Pasifik Dayanıklı Company Istanbul, specifically for Turkey and based on the German Food Code and Nutrient Data Base (Bundeslebensmittelschluessel) Version 3.01B (http://www.bfr.bund.de/cd/801) [accessed on 5 March 2022]. The analyses results were evaluated compared against RDAs, DRIs or WHO recommendations according to age, sex, and physiological status [16].

### 2.4. Determining Socioeconomic Status

Socioeconomic status was determined using TURKSTAT (Turkish Statistical Authority) reports. Distribution of annual equivalized household disposable income by quintiles ordered by equivalized household disposable income was used with 2015 values/prices (Table 1). Incomes are sorted in ascending order by equivalized household disposable income and are divided into five categories, the bottom income group is defined as “the first quintile” (lowest) and the top income group is defined as “the last quintile” (highest) [17].

These values are given for 12 months. If means are divided by 12, the first quintile is approximately 422 Turkish Lira (TL), the second 737 TL, the third 1043 TL, the fourth 1482 TL and the last quintile is 3197 TL. Mean income value is 1376 TL. For determining income levels as low, middle, and high, the previously mentioned five quintiles were condensed to three groups as equally (33%). Hence, earnings lower than 900 TL fall in the low-income category, earnings between 901–1800 TL in the middle-income category, while earnings over 1801 TL fall in the high-income category (Table 2).

While the estimates of wealth are not equivalized, the breakdown by income quintile is based on an equivalized income concept to reflect differences in household size and composition. To assign households to disposable income quintiles, equivalized household disposable income must first be estimated for each household considering distribution into components (for example compensation of employees, transfers to and from other sectors, etc.) for which corresponding variables or proxies can be found. Survey weights are considered when calculating each household’s share of each of the components. For each household, the distributed components are added up to calculate the household’s estimated disposable income. A final adjustment is done to “equivalize” the household disposable income. It consists of dividing the household disposable income by the number of consumption units for each household. This adjustment is based on the Organization of Economic Co-operation and Development (OECD)-modified equivalence scale, which assigns a value of 1 to the first adult, 0.5 to each additional person aged 14 and over, and 0.3 for all children under 14. The result is a new income variable for each household, more closely aligned with the concept of household disposable income than the available measure of after-tax income.

### 2.5. Type 2 Diabetes Mellitus Risk Assessment

Type 2 Diabetes Mellitus (T2DM) risk assessment was conducted using the Finnish Diabetes Association’s Type 2 Diabetes Risk Assessment Form (FINDRISC). The FINDRISC was established in Finland using the results of two cohort studies conducted in 1987 and 1992, respectively; these studies included 2525 participants who were followed-up for 10 years and 1976 participants who were followed up for 5 years, respectively. The maximum possible FINDRISC score is 26. The validity and reliability of the FINDRISC score in Turkish patients were previously examined, and the power of the FINDRISC score to predict newly diagnosed T2DM was 0.84 [18,19].

### 2.6. Ethics

All participants provided their informed consent for inclusion upon their recruitment in the study. The study was conducted in accordance with the Declaration of Helsinki, and the protocol was approved by the Ethics Committee/Institutional Review Board of Istanbul Yeni Yuzyil University (SBF-120705051-2016).

### 2.7. Statistical Analyses

Data were statistically analyzed with SPSS (Statistical Package for Social Sciences) for Windows 20.0 (IBM Corp, Armonk, NY, USA). Chi-square test was used for comparing classified categorical data. The Kruskal–Wallis H-test was used for three or more categorical variables, which are used for non-anthropometric data. Results were deemed statistically significant at 95% confidence level, *p* < 0.05. Post hoc Bonferroni correction was applied to the results to control for type I error due to multiple comparisons conducted, and adjusted *p*-values were produced.

## 3. Results

A total of 100 Turkish female university students constituted our participant population. Participants were either living with their family (60%) or in dormitory (40%) and were classified as per income level. More specifically, of the students living with their family 22 were low-, 15 middle- and 23 high-income. Of the 40 students living in the dormitory, 20 were low-, 10 middle-, and 10 high-income level.

According to our results income is positively correlated with physical activity, non-smoker status and non-alcohol use as well as lower T2DM risk score (Table 3). There was no difference in family history for hypertension as related to income levels, with all subgroups of participants exhibiting low prevalence of family history for hypertension. Furthermore, we did not detect differences regarding Fasting Plasma Glucose (FPG) history, T2DM risk score and family hypertension history, between student participants who resided with their parents versus those who resided in the university dormitories, thus the place of residence did not seem to affect those outcome variables (data not shown).

While income level did not have a significant effect on anthropometric parameters or T2DM risk, for the latter there was a trend observed in lower risk scores for those in the high-income level category, regardless of living status (Table 4). More specifically the FINDRISC values for low-, middle- and high-income were: 6.4 ± 3.0, 2.6 ± 2.3, 1.9 ± 1.6, respectively (Table 4). The purpose of the study was to assess how certain practices and conditions among the young college students may modulate risk for T2DM. Our results suggest that financial status inversely correlates with the risk for T2DM as judged by the FINDRISC score.

Interestingly, income status seemed to influence the pattern of BMI distribution among participants. More specifically, the higher the income, the higher the percentage of participants who were at normal BMI (Table 5). Additionally, BMI was seen to be overall higher if living in the dormitories as opposed to living with family (data not shown).

Regarding the diet and food choices of participants, our results indicate the effect of income as well as status of living in the dormitories versus with family, although the income effect seemed to be more profound. Specifically, the more expensive and healthier food choices, are selected at a significantly higher frequency by participants of higher income. Additionally, participants living with their families seemed to be making overall healthier choices in terms of foods consumed (Table 6 and Table 7). Particularly, food items such as fish, nuts, fruits and vegetables (healthier and more expensive choices) are consumed at significantly higher rates by higher income participants living with their parents (Table 6). Additionally, consumption of dairy was significantly higher as the income increased. Simultaneously, bread and pastry products are less so consumed by higher income compared to lower income participants among those who live with their family (same living status—household) (Table 6). Finally, table sugar use is notably higher for the low-income participants who live with their parents. In the case of participants living in the dormitories, we also observed the effect of income on the quality of food choices. More specifically, higher income participants demonstrated higher consumptions for dairy and chicken/turkey (Table 7), while simple and economical carbohydrate sources such as breads and pasta, as well as chocolate, were lower for this group. This observation indicated that some more expensive, yet healthier food options were more common among the participants of the higher income even though they were living in the dormitories.

From a nutritional analysis perspective, we observed that among the participants who were living with their parents there was not a significant level of income effect in terms of daily energy intake but there was an effect on macronutrient distribution as percent of energy. More specifically, higher income was not associated with lower energy intake (although there was a trend seen). A higher percent of daily energy from protein and lower from lipid was observed for the higher income compared to lower income levels (supplementary data Table S1). Micronutrient consumption (minerals and vitamins) indicated no statistically significant differences among participants regardless of financial status (level of income). For participants living in the dormitories, there was only an effect of income on protein intake with higher income significantly associated with a higher percent of daily energy from protein. No other differences were observed among participants in terms of macronutrient and micronutrient intakes (supplementary data Table S2).

In summary, our data taken together demonstrate a strong effect of income on the quality of food consumed by all participants, with the effect being more pronounced when students live with their parents. Additionally, we observed an effect of income on the distribution of the BMI, whereby higher income favors a higher representation of a normal BMI. Living conditions did not produce such an effect in the case of living with parents but did exhibit a significant effect favoring higher representation in the obese BMI for lower income (*p* = 0.018) in the participants living in the dormitories. This finding suggests that the living conditions (living with family versus in the dormitories) apparently make the effect of the financial differences (income) of the participants on BMI potentially more pronounced.

Risk factors for T2DM for the general population typically include prediabetes, overweight, 45 years or older, a parent or sibling with T2DM, physically active less than 3 times a week; for women in particular, gestational diabetes or birth to an infant over 4.1 kg. Given the aforementioned risk factors, it becomes clear that our population of study is overall at a low theoretical risk for T2DM since they are young and overall, in good general health. However, we purposely selected this population as a more conservative approach but also to illustrate how dietary habits and anthropometric characteristics may increase risk towards T2DM during the young age and formative years, thus providing a paradigm for elevated caution and preventive attitude for minimizing risk overtime. Therefore, when reading the presented results obtained it is important to remember that the comparisons are conducted within a young population and what is interesting is that even within such population it can be observed that certain lifestyle factors such as dietary choices can modulate relatively T2DM risk. In this sense it becomes more likely that diet can play an even greater role in terms of T2DM at older ages when the chronic nature of the disease becomes more evident.

## 4. Discussion

Our work presented herein was a pilot-study conducted with 100 healthy Turkish female university students in Istanbul Yeni Yuzyil University of whom 60 were living with their family (parents) and 40 were living in the university dormitories. All students were categorized according to income levels using the TURKSTAT official classification approach to investigate relationships between income, dietary habits, and type 2 diabetes risk. Dietary habits were assessed with a food frequency questionnaire and a 24 h dietary recall, while T2DM risk was assessed via FINDRISC. The main strength of the current study is the understudied setting, population, and geographic location, while the major limitations include the limited sample size and convenience mode of participant selection.

Our findings indicated that there was an inverse correlation between income and risk towards T2DM as judged by FINDRISC, regardless of living conditions (living in the dormitories versus with parents). Similar results as per the association of low income and the risk of diabetes have been reported by several studies both in Turkey [20] and elsewhere [21,22]. Moreover, research conducted in New Zealand, showed that cardiovascular disease and diabetes risk factors were more strongly associated with household income than with the individual’s occupation or education [23].

While these studies demonstrated the association of low income and higher risk for diabetes in older adults, it is interesting that the findings are similar conceptually. The arguably higher level of education in college students as well as better access to knowledge sources did not seem to alter the trend of results thus highlighting income as a critical driver of diabetes risk. This point is in accordance with research conducted in the USA as well. More specifically, in a study conducted in West Virginia University, assessing knowledge of diabetes risk factors, symptoms, and treatment/complications among college students, the authors reported that college students have misperceptions of diabetes risk despite the presence of known risk factors such as family history, overweight/obesity status, and a sedentary lifestyle, and despite their higher-than-average educational level [24]. Another study in the USA demonstrated a discordance between college students’ perceived risk and prevalence of T2DM risk factors, which warrants strategies to address misperceptions of T2DM risk and improve lifestyle behaviors among an upstate New York college student population [25].

Interestingly in our population, approximately 20% of participating students consume alcohol and a significant number of students are smokers. Our results taken through the cultural lens of Turkey agree with other reports from other researchers in similar settings in Turkey [26,27]. These observations however lend further support to the notion that a higher socioeconomic status and educational level do not necessarily always and/or fully align with healthier practices.

In our work we found that income is influencing the BMI distribution whereby healthier BMIs are seen in higher income students. More specifically, students of high-income are in normal ranges more so than students of low-income and middle-income. Low-income students have higher rates of overweight or obesity. Additionally, we did observe that BMI is overall higher in students living in the dormitories in agreement with similar work in Turkey [28]. Our findings corroborated further previous studies with similar design and setting on female university students. Our study however, to the best of our knowledge, is the first one of its kind to have been conducted on a university campus in Istanbul, the largest metropolitan area in Turkey and the main economic and trade center of the country. Collectively, these previous studies along with the present study, indicate that while overall female college students tend to be within normal BMI ranges there is an effect of income and possibly residence status for BMI [26].

In previous work in Turkey, our group demonstrated that traditional approaches could avert poor dietary habits and reduce obesity risk in children [29], while healthier eating is positively correlated with better admission scores for university entrance examinations [30]. While this earlier work included younger populations, the concept whereby living with family in a traditional society can benefit the members through a channel of overall healthier practices is something we also observed when investigating the nutritional/dietary choices of participants living in the dormitories versus those living with family. More specifically, we did observe that overall, there was a higher intake of higher quality and more nutrient-dense choices among students living with their family compared to those living in the dormitories. While income still extended an effect on diet quality, the overall nutrient intakes were better in students living with their families.

Despite notable variations, overall, there was a clear trend for higher intake for healthier and more nutrient-dense and expensive food options such as fish, meats, eggs, dairy, and nuts as well as fruits and vegetables for student participants living with their families compared to those living in the dormitories. Income remained a significant and the main driver as per the intake of higher quality nutrient-dense foods in both students living with their family and in dormitories, although even more prevalent in the former category. These findings agree with the notion that individuals of higher income tend to consume more protein than those with lower income.

In both students living with their family and in the dormitories, students of low income exhibit a higher daily caloric intake. The recommended daily energy intake for Turkish females in the 19–30-year-old age range is 2200 kcal (for 59 kg) [31]. In our population, average weight is 57.9 kg implying that inadequate energy is consumed on average. In this age group a tendency towards thinness and underweight is commonly observed in female populations [32]. A higher tendency of disordered eating was previously documented in female college students in Turkey, especially in those who do not live with their parents [33]. A higher level of disordered eating is associated with a lower health-related quality of life, subsequently leading to higher risk for developing health adversities including increased diabetes risk [33]. In a recent study conducted in Canada assessing diet quality in students living on- versus off-campus, researchers found that students living on campus experienced significantly larger gains in weight and BMI compared to students living off-campus [34]. This latter work corroborated similar earlier work in the USA, whereby it was shown that food environments in college dormitories appeared to be less than optimal in terms of health and energy density of food choices associated with on-campus living [35].

A recent extensive review of the evidence underlines the importance of the university setting in the formation of eating habits/patterns of college students and the associated potential health risks. More specifically, Bailey and colleagues document that most college students are not meeting dietary and physical activity guidelines, and the average student gains an estimated 1.6–3.0 kg during 4 years of study. Authors further present evidence supporting that campus food environments may contribute to energy overconsumption and weight gain, while the number of campuses requiring students to participate in physical activity courses is declining [36].

It is interesting however that among college students it is not uncommon to observe a low perceived risk towards chronic diseases and T2DM specifically. Research in three USA urban colleges totaling 1579 students of whom 541 were categorized as high risk for T2DM, showed that 39% of those at high risk were found to underestimate their personal risk, which may conceivably negatively affect their chances of acting preventively to reduce diabetes risk [37].

In this sense, it is important to illustrate the fact that while income and education are important towards healthier eating and lifestyles and in turn reduced risk for chronic disease, the university environment and a support system also seem to play a significant role. While similarities in trends and conclusions exist among different settings, it is also important to take into consideration the cultural norms and the customary approaches as well as social models that vary among different settings. Thus, approaches need to be tailored appropriately and consider such parameters when policies and administrative efforts at the university level are undertaken to improve the health/well-being and prosperity of students. These considerations are important since it has been shown in the Turkish setting that even relatively small interventions such as improved snacking practices can have a beneficial effect on diabetes outcomes [38]. There are studies that have indicated a potential positive role of certain macronutrients such as protein/amino acids [39] with mechanistic biological evidence for improvement on biochemical responses [40] related to progression of chronic disease including diabetes. Further investigation could reveal the feasibility of those nutrients as components for potential healthy interventions aiming at improving body composition and metabolic health.

Young adulthood, the period from late adolescence through the twenties, is associated with life transitions that could contribute to the development of obesity and/or the establishment of unhealthy patterns, routines and behaviors that increase risk for chronic diseases including T2DM. Therefore, targeting this group in a culturally appropriate manner could generate significant public health benefits through an effective disease prevention and health promotion approach. In this vein, universities could constitute promising settings to prevent the onset of unhealthy eating commonly described in the student demographic and improve nutrient intake of students [41] through multifaceted, targeted, and personalized interventions.

## 5. Conclusions

In the work presented herein, we were interested in examining whether and how the living status has an impact on female students’ nutritional habits, and how these results affect the risk of T2DM within this group, since available evidence suggests an association between income level, dietary habits, and type 2 diabetes risk, in college students. Our results showed that financial status, rather than living in the dormitories versus with family, is positively associated with increased T2DM risk among Turkish female college students as assessed via the Finnish Diabetes Association’s Type 2 Diabetes Risk Assessment Form (FINDRISC). Furthermore, our findings indicate a potential need for educational programs and nutritional support for students, particularly those living away from family.

## Figures and Tables

**Table 1:** Distribution of annual equivalized household disposable income by quintiles ordered by equivalized household disposable income for 2015 prices in Turkish Lira (TL).

	Total	First Quintile	Second Quintile	Third Quintile	Fourth Quintile	Last Quintile
%	100	6.1	10.7	15.2	21.5	46.5
Mean (TL)	16,515	5065	8850	12,520	17,785	38,368
Median (TL)	12,492	5306	8812	12,492	17,558	30,993

**Table 2:** Cut-offs for low-, middle-, high-income classification from quintiles (values pertain to monthly net).

Classification	TL
Low-income	<900
Middle-income	901–1800
High-income	>1801

**Table 3:** Participant characteristics analyzed according to income.

Income Level									
	Low Income (n = 42)		Middle Income (n = 33)		High Income (n = 25)		Total (n = 100)		*p*
Status	N	%	N	%	N	%	N	%	
*Living*									0.945
Family	22	52.4	23	69.7	15	60	60	60	
Dormitory	20	47.6	10	30.3	10	40	40	40	
*Smoking*									0.002
Yes	2	4.8	5	15.2	16	64	23	23	
No	40	95.2	28	84.8	9	36	77	77	
*Alcohol*									0.001
Yes	1	2.4	8	24.2	11	44	20	20	
No	41	97.6	25	75.8	14	56	80	80	
*Physical Activity*									0.002
Yes	14	33.3	31	93.9	25	100	70	70	
No	28	66.7	2	6.1	0	0	30	30	
*High FPG History*									0.002
Yes	35	83.3	15	45.5	7	28	57	57	
No	7	16.7	18	54.5	18	72	43	43	
*Hypertension History*									0.354
Yes	3	7.1	0	0	0	0	3	3	
No	39	92.9	33	100	25	100	97	97	
*T2DM risk (FINDRISC)*									0.002
Low (<7)	23	54.8	30	90.9	24	96	77	77	
Slightly elevated (7–11)	17	40.5	3	9.3	1	4	21	21	
Moderate (12–14)	2	4.8	0	0	0	0	2	2	

*p*-values were calculated via chi square test, and post hoc corrected via Bonferroni correction, thus adjusted *p*-values are presented here. Significance was accepted at *p* < 0.05.

**Table 4:** Anthropometric measurements and diabetes risk score (FINDRISC) according to income.

Income Level									
Anthropometric Measurements	Low Income (n = 42)			Middle Income (n = 33)			High Income (n = 25)		
	$\bar{x}$ ± SD	Med	L-U	$\bar{x}$ ± SD	Med	L-U	$\bar{x}$ ± SD	Med	L-U
T2DM risk (FINDRISC)	6.4 ± 3.0	5.5	0–12	2.6 ± 2.3	3	0–11	1.9 ± 1.6	1	0–8
Weight (kg)	61.3 ± 10.2	60	43–80	55.3 ± 9.5	54	45–98	57.2 ± 6.3	56	49–70
Waist Circumference (cm)	75.0 ± 14.5	74.5	65–96	72.1 ± 8.8	70	63–108	73.3 ± 6.5	70	65–90
Height (cm)	164.5 ± 6.4	165	150–180	163.6 ± 4.1	165	155–170	164.5 ± 4.4	165	156–172
BMI	22.7 ± 3.6	23	16–33	20.8 ± 3.9	19.9	17–38	21.2 ± 2.1	20.9	17–26

Med: Median; L-U: Lower-Upper (range); SD: Standard Deviation of the mean; $\bar{x}$: mean.

**Table 5:** Classification of BMI according to income level.

Income Level									
	Low Income (n = 42)		Middle Income (n = 33)		High Income (n = 25)		Total (n = 100)		*p*
BMI Classification	N	%	N	%	N	%	N	%	
									0.015
Underweight (≤18.4)	7	17	10	30	1	4	18	18	
Normal weight (18.5–24.9)	22	52	21	64	22	88	65	65	
Overweight (25.0–29.9)	12	29	1	3	2	8	15	15	
Obese (≥30.0)	1	2	1	3	0	0	2	2	

*p*-value is calculated via chi square test, and post hoc corrected via Bonferroni correction, thus the adjusted *p*-value is presented here.

**Table 6:** Food group consumption in students living with parents stratified by income.

Income Level									
	Low Income (n = 22)		Middle Income (n = 23)		High Income (n = 15)		Total (n = 60)		*p*
Foods (g/day)	$\bar{x}$ ± SD	L-U	$\bar{x}$ ± SD	L-U	$\bar{x}$ ± SD	L-U	$\bar{x}$ ± SD	L-U	
*Milk and Dairy*									
Milk	71.7 ± 24.7	23–160	100.4 ± 38.7	69–160	125.7 ± 40.8	69–160	96.2 ± 40.2	23–160	0.003
Yoghurt–ayran–kefir	109.8 ± 67.9	29–200	149.1 ± 60.9	29–200	222.9 ± 114.3	57–400	153.1 ± 89.8	29–400	0.024
Cheese varieties	28.6 ± 13.6	10–70	45.2 ± 16.1	30–70	48 ± 17.4	30–70	39.8 ± 17.6	10–70	0.002
*Meat–Egg–Legume*									
Red Meat	15.3 ± 6.2	0–25	8.4 ± 7.6	0–20	11.8 ± 8.4	0–25	31.8 ± 25.1	0–120	0.045
Chicken–turkey	16.9 ± 8.7	9–29	26.9 ± 18.7	9–86	50.5 ± 23.5	14–100	29.1 ± 21.4	9–100	0.003
Fish varieties	24.5 ± 19.2	0–69	29.8 ± 19.2	0–57	47.9 ± 25.6	4–86	32.3 ± 22.6	0–86	0.036
Egg	34.4 ± 12.4	14–50	36.5 ± 17.0	0–50	41.9 ± 14.5	0–50	37.1 ± 14.9	0–50	0.324
Legumes	72.7 ± 41.6	0–171	83.4 ± 46.1	13–171	91.4 ± 52.8	43–200	81.5 ± 46.1	0–200	1.470
Nuts	15.9 ± 8.9	0–34	19.4 ± 8.8	11–40	30.3 ± 10.6	11–40	20.8 ± 10.8	0–40	0.003
*Fruits*	156.3 ± 63.7	86–300	216.2 ± 92.3	86–450	274.3 ± 52.6	150–300	208.7 ± 86.1	86–450	0.002
*Vegetables*	71.7 ± 24.7	23–160	100.4 ± 38.7	69–160	125.7 ± 40.7	69–160	96.3 ± 40.3	23–160	0.002
*Bread and Cereals*									
Bread	150.2 ± 51.1	12–210	157.5 ± 54.1	14–210	131.7 ± 29.6	43–150	148.4 ± 48.3	12–210	0.063
Pilaf–Bulgur–Pasta	92.2 ± 45.0	29–200	64.0 ± 30.4	29–129	60.3 ± 34.5	5–100	73.4 ± 39.5	5–200	0.222
Pastry Products	91.8 ± 45.0	29–150	55.6 ± 36.2	7–129	35.1 ± 30.6	7–129	63.7 ± 44.2	7–150	0.002
*Sugar and Desserts*									
Chocolate	21.7 ± 10.6	6–40	16.4 ± 9.5	6–40	14.7 ± 10.1	1–40	17.9 ± 10.3	1–40	0.456
Honey–jam–pekmez	9.3 ± 4.7	0–15	8.9 ± 5.7	0–15	8.5 ± 4.7	1–15	8.9 ± 5.0	0–15	2.718
Table Sugar	15.3 ± 6.2	0–25	8.4 ± 7.6	0–20	11.8 ± 8.4	0–25	11.7 ± 7.8	0–25	0.045

The *p*-values are calculated with Kruskal–Wallis H-Test and independently from the daily energy consumption. *p*-values were post hoc corrected via Bonferroni correction, thus adjusted *p*-values are presented here. Statistical significance was accepted at *p* < 0.05.

**Table 7:** Food group consumption in students living in the dormitories stratified by income.

Income Level									
	Low Income (n = 22)		Middle Income (n = 23)		High Income (n = 15)		Total (n = 60)		*p*
Foods (g/day)	$\bar{x}$ ± SD	L-U	$\bar{x}$ ± SD	L-U	$\bar{x}$ ± SD	L-U	$\bar{x}$ ± SD	L-U	
*Milk and Dairy*									
Milk	44.0 ± 21.1	11–80	58.3 ± 32.5	34–137	54.3 ± 22.2	23–86	50.1 ± 24.8	11–137	1.518
Yoghurt–ayran–kefir	82.8 ± 39.2	0–171	134.3 ± 50.4	57–200	174.3 ± 34.2	114–200	118.6 ± 55.8	0–200	0.002
Cheese varieties	12.6 ± 6.9	0–30	20.0 ± 8.2	10–40	21.5 ± 9.4	10–35	16.7 ± 8.7	0–40	0.033
*Meat–Egg–Legume*									
Red Meat	13.4 ± 5.9	0–21	11.1 ± 6.5	0–20	10.3 ± 4.2	4–17	35.5 ± 27.5	0–90	0.558
Chicken–turkey	18.4 ± 7.9	7–29	40.7 ± 19.7	7–57	55.0 ± 18.4	29–86	33.1 ± 21.1	7–86	0.002
Fish varieties	9.4 ± 7.8	0–29	14.8 ± 12.2	0–43	16.5 ± 18.5	0–57	12.5 ± 12.4	0–57	0.885
Egg	18.0 ± 9.9	0–43	25.7 ± 6.0	14–29	29.3 ± 14.4	14–50	22.8 ± 11.4	0–50	0.084
Legumes	37.5 ± 17.8	7–86	48.1 ± 22.2	10–86	56.7 ± 26.9	10–86	44.9 ± 22.4	7–86	0.246
Nuts	16.0 ± 10.5	0–40	15.7 ± 11.2	0–34	20.0 ± 11.5	11–40	16.9 ± 10.8	0–40	1.635
Fruits	55.0 ± 22.4	29–86	72.1 ± 41.8	29–150	67.1 ± 24.3	29–86	62.3 ± 29.0	29–150	1.197
Vegetables	44.0 ± 21.1	11–80	58.3 ± 32.5	34–137	54.3 ± 22.2	23–86	50.1 ± 24.8	11–137	1.518
*Bread and Cereals*									
Bread	119.4 ± 39.2	10–180	143.1 ± 30.1	86–180	53.1 ± 19.2	26–86	108.8 ± 46.9	10–180	0.003
Pilaf–Bulgur–Pasta	77.9 ± 20.5	29–100	72.9 ± 28.1	29–100	45.7 ± 14.7	29–57	68.6 ± 24.9	29–100	0.012
Pastry Products	80.7 ± 32.9	29–129	85.7 ± 26.1	57–129	62.9 ± 26.2	29–86	77.5 ± 30.3	29–129	0.807
*Sugar and Desserts*									
Chocolate	27.6 ± 9.2	11–40	15.1 ± 8.3	3–23	14.9 ± 3.7	9–20	21.3 ± 10.1	3–40	0.003
Honey–jam–pekmez	5.1 ± 2.9	0–9	4.7 ± 4.1	1–13	5.6 ± 4.2	1–15	5.1 ± 3.5	0–15	2.457
Table Sugar	13.4 ± 5.9	0–21	11.1 ± 6.6	0–20	10.3 ± 4.2	4–17	12.0 ± 5.7	0–21	0.558

The *p*-values are calculated with Kruskal–Wallis H-Test and independently from the daily energy consumption. *p*-values were post hoc corrected via Bonferroni correction, thus adjusted *p*-values are presented here. Statistical significance was accepted at *p* < 0.05.

## Data Availability

Data are available by the corresponding author as needed.

## Supplementary Materials

The following supporting information can be downloaded at: https://www.mdpi.com/article/10.3390/bs12090309/s1. Table S1: Energy and Nutrient Intake of Students According to Income (living with their family) (kcal/day and g/day), Table S2: Energy and Nutrient Intake of Students According to Income (living in dormitory) (kcal/day and g/day), Questionnaire used (versions: Turkish and English).
