# Supplementary material for: The Risk of T2DM in College Women: The Predictive Power of Financial versus Residential Status in a Cross-Sectional Pilot Study in Turkey
Source: Behav Sci (Basel). 2022 Aug 26;12(9):309. doi: 10.3390/bs12090309 (PMC9495967; doi:10.3390/bs12090309)
Supplement: Supplementary file 1 [file behavsci-12-00309-s001.zip › behavsci-1763510-supplementary.pdf]

Supplemental materials

Table S1. Energy and Nutrient Intake of Students According to Income (living with their family) (kcal/day and g/day).

	**Income Level**												
	**Low Income (n=20)**			**Middle Income (n=10)**			**High Income (n=10)**			**Total (n=40)**			**p**
**Amount of Energy and Nutrients**	**X ± SD**	**Med**	**L-U**	**X ± SD**	**Med**	**L-U**	**X ± SD**	**Med**	**L-U**	**X ± SD**	**Med**	**L-U**	
Energy (kcal)	1364.5±164.6	1372.5	1057-1594	1287.1±295	1286	820-2206	1184.3±172.5	1183	922-1578	1289.8±232.6	1280.5	820-2206	0.078
Protein (g)	47.9±16.5	45	28-110	54.7±17.9	52.8	24-97	55.8±13.3	50.7	40-91	52.5±16.5	50.2	24-110	0.186
Protein (%)	14.0±4	14	8-29	17.1±3.9	16	12-27	19.2±2.7	19	15-24	16.5±4.2	16	8-29	0.002*
Fat (g)	53.2±9.6	55.6	37-71	46.4±14.9	47.3	22-86	39.5±8.7	38.5	25-59	47.7±12.8	46.2	22-86	0.003*
Fat (%)	34.5±3.7	36	29-41	31.9±4.3	33	20-36	29.3±3	30	23-33	31.8±4.3	32	20-41	0.002*
Carbohydrate (g)	168.6 ±23	178.4	114-196	162.7 ±31.2	153.7	124-265	146.6 ±17.3	149	105-171	160.8 ±26.5	159.2	105-265	0.050*
Carbohydrate (%)	51.4±6.3	51	30-58	51.9±5.2	51	43-62	51.7±4.5	51	44-60	51.7±5.4	51	30-62	2.862
Dietary Fiber (g)	14.8±4.8	15.5	7-23	15.5±4.5	15	6-24	14.0±3.8	13.3	11-26	14.9±4.4	14.1	6-26	1.188
PUFA (g)	17.4±32.4	10.2	5-161	8.8±3.9	8.9	2-18	8.1±4.9	7.7	3-22	11.7±20.1	8.8	2-161	0.426
Cholesterol (mg)	177.7±104.4	163.8	32-377	196.9±100	208.8	39-391	184.4±93.2	185.1	50-318	186.7±98.7	188.1	32-391	2.448
Vitamin A (µg)	1451.7±1590.1	1046.3	309-649	920.8±566.6	929.6	0-1903	807.1±460.7	679	141-1614	1087±1072	913.5	0-6499	0.990
Carotene (mg)	3.6±3.1	2.7	1-13	2.7±1.8	2.3	0-6	2.2 ±1.6	1.6	0-5	2.9 ±2.5	2.3	0-13	1.194
Vitamin E (mg)	10.3±4.7	9	3-23	9.5±4.3	9.6	2-19	8.2±5.1	7.4	3-23	9.4±4.6	8.9	2-23	0.657
Vitamin B1 (mg)	0.5±0.2	0.5	0-1	0.6±0.2	0.5	0-1	0.6±0.2	0.5	0-1	0.6±0.2	0.5	0-1	1.422
Vitamin B2 (mg)	0.8±0.3	0.8	0-2	0.9±0.3	1	0-1	0.9±0.3	0.8	1-2	0.9±0.3	0.8	0-2	0.843
Vitamin B6 (mg)	0.8±0.3	0.8	0-1	0.9±0.3	0.8	1-2	0.8±0.3	0.7	1-1	0.8±0.3	0.8	0-2	2.310
Tot. Folic acid (µg)	161.8±47.5	151.3	86-234	185.8±58.8	187	99-298	183.1±48.2	173	124-306	176.4±52.6	173.3	86-306	1.140
Vitamin C (mg)	69.7±53	51.6	0-171	80.6±47	76.5	1-178	58.8±34.5	47.5	14-122	71.2±46.7	56.7	0-178	0.885
Sodium (mg)	2543.6±896.9	2350.5	491-3816	2999±1001	3060	1329-5878	2784±691	2688	1154-3795	2778.5±902.1	2891	491-5878	1.302
Potassium (mg)	1507.5±583.1	1300	518-2610	1633.6±597.8	1660	658-2958	1398±465	1444	735-2507	1528.5±560.8	1462	518-2958	1.500
Calcium (mg)	491.8±240.2	504.1	122-990	541.1±192.7	536	117-982	469.9±267.8	437	119-1066	505.2±228.6	504	117-1066	1.005
Magnesium (mg)	164.2±51	163.8	70-257	169.9±45	176	91-270	169.8±64.1	140	99-308	167.8±51.6	162	70-308	2.601
Phosphorus (mg)	707.3±206.2	724	412-1185	836.2±199.9	829	389-1251	755.9±232.7	716	472-1392	768.8±214.7	752	389-1392	0.138
Iron (mg)	7.1±1.9	6.9	3-11	7.75±2.6	7.8	0-12	7.7±1.4	7.7	6-12	7.5±2.1	7.4	0-12	1.026
Zinc (mg)	5.8±1.7	5.9	2-9	7.7±2.7	7.7	3-14	7.4±2.2	7.2	4-11	6.9±2.4	6.7	2-14	0.084

-p values are calculated with Kruskal Wallis H-Test; p values are calculated independently from the daily energy intake. P values presented here are adjusted after post hoc Bonferroni correction. * $p < 0.05$.

Table S2. Energy and Nutrient Intake of Students According to Income (living in dormitory) (kcal/day and g/day).

	Income Level												
	**Low Income (n=20)**			**Middle Income (n=10)**			**High Income (n=10)**			**Total (n=40)**			**p**
**Amount of Energy and Nutrients**	***X*** **± SD**	**Med**	**L-U**	***X*** **± SD**	**Med**	**L-U**	***X*** **± SD**	**Med**	**L-U**	***X*** **± SD**	**Med**	**L-U**	
Energy (kcal)	1324.5±321	1360	810-1861	1210.5±293.6	1249.5	542-1656	1247.4±287.7	1248	801-1703	1276.7±302.9	1280.5	542-1861	2.013
Protein (g)	45.4±16	43.5	23-76	54.8±11.2	57.2	27-67	67.9±14.8	63.1	49-95	53.4±17	56.5	23-95	0.015*
Protein (%)	13,65±3	13	10-22	18.6±1.5	19.0	17-21	22.6±5.3	21	18-34	17.1±5.1	17	10-34	0.002*
Fat (g)	62±22.2	63.9	15-105	54.1±19.2	52.9	10-82	51.1±24.1	48.5	12-85	57.3±22	58.9	10-105	1.278
Fat (%)	40.1±9.1	41.5	14-52	38.2±8.9	40.5	16-46	34.3±11.1	37.5	13-46	38.2±9.6	40.5	13-52	0.486
Carbohydrate (g)	148.5±32.4	158.7	60-189	241.6±376.7	119.3	85-1311	128.5±17.1	122.1	108-153	166.8±187.9	139.8	60-1311	0.174
Carbohydrate (%)	46.2±9.6	44	33-76	43.1±8.6	39.5	35-64	43±7.7	42.5	30-58	44.7±8.8	43	30-76	0.873
Dietary Fiber (g)	14.1±5.8	13	2-24	11.4±3.1	10.2	7-17	13.3±4.3	13.3	7-20	13.3±4.9	12.4	2-24	0.846
PUFA (g)	12.8±6	13.4	3-23	11.6±4.1	12.1	2-16	11.3±6.4	12.2	2-25	12.2±5.6	12.8	2-25	2.118
Cholesterol (mg)	378.5±124.5	376.8	120-828	300.3±122.4	305.4	87-502	291.7±125.1	248.7	157-551	337.3±128	352.5	87-828	0.204
Vitamin A (µg)	838.7±399.9	855.3	110-1602	667.6±272.8	601.2	312-1023	854.9±452.5	966.4	3-1536	800±385.2	836.7	3-1602	1.116
Carotene (mg)	2.1±1.7	1.5	0-6	1.3±0.8	1.2	0-3	3.9±4.7	3.2	1-17	2.3±2.7	1.7	0-17	0.489
Vitamin E (mg)	12.1±5.8	12.3	3-25	10.1±4.3	10.9	3-16	9.8±4.6	10.6	3-17	11.1±5.2	11.5	3-25	1.383
Vitamin B1 (mg)	0.5±0.2	0.5	0-1	0.5±0.1	0.5	0-1	0.6±0.2	0.5	0-1	0.6±0.2	0.5	0-1	2.850
Vitamin B2 (mg)	0.9±0.3	0.8	0-2	0.9±0.3	0.9	0-1	0.8±0.3	0.8	1-2	0.9±0.3	0.8	0-2	1.458
Vitamin B6 (mg)	0.8±0.2	0.8	0-1	0.8±0.2	0.7	0-1	1.4±1.3	1.2	1-5	0.9±0.7	0.8	0-5	0.099
Tot. Folic acid (µg)	175.2±59.7	166.4	82-303	152.4±41.9	155.6	88-209	166.5±60.9	156.8	87-291	167.3±55.5	160	82-303	1.947
Vitamin C (mg)	66.1±58.9	51.6	0-217	49.9±42.7	38	0-156	54.3±29.8	43.1	17-114	59.1±48.7	43.5	0-217	2.340
Sodium (mg)	3603.9±595.4	3770.2	2208-4667	3416±1167	3594.5	902-5035	3271±1074	3458	1586-5369	3473.7±879.6	3625	902-5369	1.053
Potassium (mg)	1439.5±564.2	1322.5	322-2748	1356.3±545.5	1388	591-2335	1515±478	1442	840-2356	1437.7±528.9	1388	322-2748	2.580
Calcium (mg)	458.4±206.7	438.4	36-972	452.1±214.8	481.6	94-744	412.8±240.7	308.5	158-900	445.4±212.6	387	36-972	1.665
Magnesium (mg)	177.7±78.1	166.5	59-387	146.7±47.2	159.3	57-210	180.6±49.1	165.9	124-268	170.7±65.1	163	57-387	1.500
Phosphorus (mg)	741.6±268.5	686	300-1204	773.9±170.6	794.4	394-976	849.2±237	813.3	566-1251	776.6±238.3	767	300-1251	1.575
Iron (mg)	7.3±2.4	6.9	2-11	7.4±1.3	7.9	5-9	8.3±1.9	8.2	6-11	7.6±2.1	7.4	2-11	1.536
Zinc (mg)	6.7±3.2	5.9	2-14	8.3±2.3	8.6	4-11	8.2±3.4	8.5	4-15	7.5±3.1	6.6	2-15	0.420

-p values are calculated with Kruskal Wallis H-Test; p values are calculated independently from the daily energy intake. P values presented here are adjusted after post hoc Bonferroni correction. * $p < 0.05$.

English Version of Survey

SURVEY FOR DETERMINING RELATIONSHIPS BETWEEN SOCIECONOMIC STATUS, DIETARY HABITS AND RISK OF TYPE II DIABETES IN FEMALE STUDENTS WHO ARE LIVING WITH THEIR FAMILY OR IN THE DORMITORY

This research will be used for determining relationships between socioeconomic status, dietary habits and the risk of Type II diabetes on female Turkish university students who live with their family or in dormitory. Participating in this survey is on voluntary basis. Thank you for your time and answers.

1)I am living with my family / in dormitory. (please circle the most accurate)

2)Age:

3)Weight :

4)Height :

5)Waist circumference :

6)Do you have a mother, father of brother with diabetes ? 1.YES / 2. NO

7) Do you have a grandparent with diabetes? 1.YES / 2.NO

8) Have you ever been diagnosed with high blood pressure ? 1.YES / 2.NO

9) Do you physically active ? (at least 30 min. in a day) 1. YES / 2.NO

10) Do you smoke? 1. YES / 2. NO

11) Are you using alcohol ? 1. YES / 2. NO

12) Level of income :

1. Less than 900 TL 2. Between 901- 1800 TL 3. More than 1801 TL

Food Frequency Questionnaire

FOOD/FOOD GROUPS	EVERY DAY	1-2 TIMES /WEEK	3-4 TIMES /WEEK	5-6 TIMES /WEEK	2 DAYS /MONTH	Once/ MONTH	NEVER CONSUME	AMOUNT
**MILK/MILK PRODUCTS**								
Milk								
Yoghurt,airan,kefir								
Cheese varieties								
**MEAT PRODUCTS/ EGG/ LEGUMES**								
Red meat								
Chicken,turkey								
Fish								
Egg								
Legumes								
Nuts (walnut, almond etc.)								
**FRUITS**								
**VEGETABLES**								
**GRAINS**								
Bread								
Rice,pasta,bulgur								
Cookies,cake and other pastry products								
**SUGAR/DESSERTS**								
Chocolate								
Honey-jam-pekmez								
Table sugar								

24 Hour Dietary Recall

Please write below what you consumed yesterday.

	NAME/TYPE OF MEAL	AMOUNT
BREAKFAST		
1. BREAK/SNACK		
LUNCH		
2. BREAK/SNACK		
DINNER		
IN-BETWEEN (OTHER)		

Turkish Version of Survey (Original)

AİLESİYLE VEYA YURTTA YAŞAYAN ÜNİVERSİTE ÖĞRENCİLERİNDE BESLENME ALIŞKANLIKLARINA ve SOSYOEKONOMİK DURUMA GÖRE TİP 2 DİYABET RİSKİ HESAPLANMASI ANKETİ

Bu araştırma ailesiyle veya yurtta yaşayan, üniversite eğitimi gören kız öğrencilerde, beslenme alışkanlıklarına ve sosyoekonomik duruma göre tip 2 diyabet riskinin hesaplanmasına dayanmaktadır. Vereceğiniz cevaplar araştırma sonucunun güvenilirliği için oldukça önem taşımaktadır. Bu ankete katılmak tamamen gönüllülük esaslarına dayanmaktadır. Zamanınızı ayırdığınız için teşekkür ederim…

1)Ailemle yaşıyorum. / Yurtta yaşıyorum. (sizin için uygun olanı yuvarlak içine alınız)

2)Yaş :

3)Kilo:

4)Boy:

5)Bel çevresi :

6)Ailenizde herhangi 1. Derece akrabanızda (anne, baba, kardeş gibi) diyabet var mı ? 1.EVET / 2.HAYIR

7)Ailenizde büyükbaba,büyükanne gibi akrabanızda diyabet var mı ? 1. EVET / 2. HAYIR

8)Bu güne kadar hipertansiyon(yüksek tansiyon) tanısı aldınız?

1. EVET / 2.HAYIR

9)Günde en az 30 dakika fiziksel aktivite yapıyor musunuz? 1. EVET / 2.HAYIR

10)Sigara içiyor musunuz? 1. EVET / 2. HAYIR

11)Alkol kullanıyor musunuz? 1. EVET / 2. HAYIR

12) Gelir düzeyiniz :

1. 900 TL'den az 2. 901 TL-1800 TL arası 3. 1801 TL'den fazla

BESİN TÜKETİM SIKLIĞI ANKETİ

**BESİN GRUBU**	HER GÜN	HAFTADA 1-2 GÜN	HAFTADA 3-4 GÜN	HAFTADA 5-6 GÜN	AYDA 2 GÜN	AYDA 1 GÜN	ASLA TÜKETMEM	MİKTAR
**SÜT/SÜT ÜRÜNLERİ**								
Süt								
Yoğurt,ayran,kefir								
Peynir çeşitleri								
**ET ÜRÜNLERİ/YUMURTA/ KURUBAKLAGİL**								
Kırmızı et								
Tavuk,hindi								
balık								
yumurta								
Kurubaklagil(nohut,kuru Fasülye vb.)								
Kuruyemiş (badem, fındık, ceviz vb.)								
**MEYVE**								
**SEBZE**								
**EKMEK-TAHILLAR**								
Ekmek								
Pilav,bulgur,makarna								
Hamur işleri(kek, börek, kurabiye vb.)								
**ŞEKER/TATLILAR**								
Çikolata								
Bal-reçel-pekmez								
kesme şeker								

1 GÜNLÜK BESİN TÜKETİM FORMU

Dün neler yiyip içtiğinizi miktarları ile belirtiniz.

	ÖĞÜN	MİKTAR
KAHVALTI		
ARA ÖĞÜN		
ÖĞLE YEMEĞİ		
ARA ÖĞÜN		
AKŞAM YEMEĞİ		
ARA ÖĞÜN		
